# Access to communication technologies in a sample of cancer patients: an urban and rural survey

**DOI:** 10.1186/1471-2407-5-18

**Published:** 2005-02-17

**Authors:** Ma'n Abdullah, Dale E Theobald, Donna Butler, Kurt Kroenke, Anthony Perkins, Sara Edgerton, William M Dugan

**Affiliations:** 1 Regenstrief Institute for Health Care, Department of Medicine, Indiana University School of Medicine and the Richard L. Roudebush VA Medical Center, Indianapolis, IN, USA; 2 Community Cancer Care, Community Health Network, Indianapolis, IN, USA

## Abstract

**Background:**

There is a growing awareness among providers of the symptom burden experienced by cancer patients. Systematic symptom screening is difficult. Our plan was to evaluate a technology-based symptom screening process using touch-tone telephone and Internet in our rural outreach cancer program in Indiana. Would rural patients have adequate access to technologies for home-based symptom reporting?

**Objectives:**

1) To determine access to touch-tone telephone service and Internet for patients in urban and rural clinics; 2) to determine barriers to access; 3) to determine willingness to use technology for home-based symptom reporting.

**Methods:**

Patients from representative clinics (seven rural and three urban) in our network were surveyed. Inclusion criteria were age greater than 18, able to read, and diagnosis of malignancy.

**Results:**

The response rate was 97%. Of 416 patients completing the survey (230 rural, 186 urban), 95% had access to touch-tone telephone service, while 46% had Internet access (56% of urban patients, 38% of rural patients). Higher rates of Internet access were related to younger patient age, current employment, and higher education and income. The primary barrier to Internet access was lack of interest. Use of the Internet for health related activities was less than 50%. The preferred means of symptom reporting in patients with internet access were the touch-tone telephone (70%), compared to reporting by the Internet (28%).

**Conclusion:**

Access to communication technologies appears adequate for home-based symptom reporting. The use of touch-tone telephone and Internet reporting, based upon patient preference, has the potential of enhancing symptom detection among cancer patients that is not dependent solely upon clinic visits and clinician inquiry.

## Background

In recent years awareness of the symptom burden experienced by many cancer patients has grown [1,2]. At some time in their illness, symptoms such as fatigue, pain, nausea, depression, and hopelessness are very likely to occur. These symptoms can be disabling and they can even limit treatment. There is a growing body of literature demonstrating that interventions for these troubling symptoms are effective [3,4]. These interventions can improve the patient's quality of life by enabling the patient to function better at home and at work.

While there is awareness among providers of symptom distress experienced by patients and there are effective symptom interventions, the problem in the day-to-day care of cancer patients is symptom identification [5]. At a recent meeting convened by the National Institute of Health, it was concluded that little is known about the actual frequency and validity of symptom screening for common cancer and cancer treatment related symptoms. In the summary statement there was expert consensus about the need for routine screening for symptoms from the point of diagnosis. Assessments should be repeated during the course of treatment. Symptom data should be integrated into routine care of cancer patients.

Community Cancer Care (CCC) is an organization with home offices in Indianapolis, Indiana, that provides professional services and program development services to 23 hospitals throughout the state of Indiana. Professional oncology services are provided by 18 medical oncologists-hematologists who are employed by CCC or serve under contract. One psychiatrist, an advanced-practice nurse, and a certified nurse are dedicated to quality-of-life efforts. Each year, an average of 2500 new patients are seen in the network of clinics. At any given time, approximately 16,000 patients are receiving care in the CCC network. While the CCC has clinics in metropolitan Indianapolis, rural outreach and program development in rural hospitals have been a major focus of CCC since its inception in 1983. Twenty-one clinics are located in Indiana towns with populations less that 16,000. Twenty counties served by CCC have populations less than 45,000.

Using paper and pencil scales we unsuccessfully tried to install a symptom screening process into the daily clinic workflow. The clinic process was slowed. Some patients could not complete the instruments. Patients' report of their symptoms could not be analyzed quickly and placed on the chart for the provider to use. Symptom screening was limited to the day of the clinic visit. We could not easily evaluate a patient's status between office visits. Trends in symptom occurrence were difficult to identify. With pencil and paper instruments it was a laborious and expensive process to establish a database for our patients' symptom reports, a necessary step in program evaluation.

Because of these limitations, our goal is to develop a technology solution to gather, analyze, and present symptom reports to physicians and nurses. Several feasible options for reporting symptoms would include either a touch-tone telephone or an Internet connected computer. Because of well-documented differences between access to the telephone service and the Internet [7], we conducted a survey in urban and rural oncology clinics to determine how many of our network patients had access to the required communication technology. For patients who had access to the Internet we were interested in identifying predictors of access as well as patients' willingness to use the Internet for symptom reporting and other cancer-related reasons.

## Methods

### Procedures

The study design and survey instrument were reviewed and approved by the Institutional Review Board of Community Medical Research Institute in Indianapolis.

A convenience sample of cancer patients was gathered from the clinic network of CCC. Three urban clinics and seven rural clinics were conveniently selected for data collection. All of these sites had concentrated, busy clinic days during which patients could be recruited. Clinics were designated "urban" or "rural" based on their zip code being categorized urban or rural by the U.S. Department of Health and Human Services, Office of Rural Health Policy [6].

Staff members at clinic sites were instructed to offer the survey instrument to all patients attending clinic during selected weeks of March and April 2003. All patients were volunteers. All patients had to be at least 18 years of age, be able to read, and have a diagnosis of malignancy (either solid tumor or blood). The number of patients who refused to complete the survey was recorded.

### The survey instrument

The survey instrument included nine items about demographics and access to touch tone telephone service and the Internet. If patients indicated they did not have access to the Internet the survey instrument directed them to questions about reasons they did not have access. If patients indicated they did have access to the Internet, the survey instrument directed them to seven additional questions about how they use or might use the Internet.

### Statistical analysis

We used two-sample t-tests to test for mean differences and chi-square tests to test for differences in proportions of demographic characteristics across clinic setting and access to the Internet. Logistic regression models were used to evaluate access to the Internet as a function of clinic setting adjusting for demographic characteristics.

## Results

Four hundred and sixteen patients completed the survey (230 rural, 186 urban). The response rate was 97%. Thirteen patients refused to complete the survey stating they were too ill or too tired. Table 1 summarizes characteristics of the sample, comparing patients in urban vs. rural settings. Patients in the rural sample were significantly older, had lower education levels, and were more likely to be Caucasian than patients in the urban sample. Touch-tone telephone service was available to most (95%) respondents, while 46% (95% [CI] 0.41–0.51), had access to the Internet. Compared to urban patients, those in rural settings had comparable telephone access but were less likely to have Internet access (38% vs. 56%, p < .001). Most patients (> 80%) reported accessing e-mail and Internet from home. As shown in Table 2, patients with Internet access were significantly younger and had higher education and income levels than patients without Internet access. Additionally, patients with Internet access were more likely to be currently employed and from an urban clinic. Table 3 summarizes the results of a logistic regression model for Internet access. Higher income and current employment increased the likelihood of having Internet access while older age and less education decreased the likelihood.

Two-thirds (67%) of people cited lack of interest for not having Internet access. Other common reasons were unfamiliarity with the Internet (21%), cost (20%), and hesitation to use a computer (13%). There were no significant differences between the urban and rural patients regarding why they did not access the Internet.

Fifty percent of patients with Internet access reported using it for health care purposes in both rural and urban clinics, and nearly 60% reported having used the Internet to seek information about their cancer. Among the 169 patients with Internet access who indicated their preferred method(s) for symptom reporting, the telephone was identified as the most popular method (70.4% of respondents) followed by Internet-based symptom reporting (28%) and touch-screen computer in the clinic waiting room (15%). Compared to urban patients, rural patients were somewhat more likely to prefer telephone symptom reporting (79% vs. 63%, P = .02) and less likely to prefer Internet bases reporting (20% vs. 35%, P = .02).

Finally, 137 respondents indicated the different ways they might use the Internet for their health care. Requesting information from a physician or nurse was the most frequently cited potential use (77% of respondents). Other reasons included, submitting information about their own condition (59%), identifying and managing symptoms (54%), scheduling appointments (52%), and obtaining prescriptions (50%).

## Discussion

The high rate (95%) of access to touch-tone telephone service among cancer patients in our network is comparable to data from other government surveys [7,8]. Internet access in both our urban sample (56%) and our rural sample (38%) are below general population estimates for the United States [9], but equal to the data generated for Indiana in a 2000 survey [7]. In a more recent survey, 63% of Indiana residents reported access to the Internet [10]. While the proportion reporting access in our sample was less, this may in part be due to the over-sampling of rural subjects as well as certain demographic characteristics. Age, education level, income, and employment status were major variables influencing Internet access. While fewer individuals in rural seetings reported having internet access, the rural-urban differences were no longer significant when adjusting for age, educational level, annual income and employment status. Thus rural-urban differences may be due to socio-demographic factors more than to a higher presence of technology barriers in rural settings.

Barriers to Internet use identified by patients and limited use of the Internet offer opportunities for better patient communication and education. Over half of the patients without Internet access reported they were not interested. Perhaps waiting room computers with links to cancer-related web sites with good educational and problem-solving content could spur interest. Educational programs for our cancer patients about the Internet and its use may also be helpful. Cost of Internet services did not seem to be a significant factor.

These data suggest that a very significant proportion of cancer patients (more that half of those with Internet access) were willing to use this modality to communicate with their cancer clinic for multiple tasks. While email may offer a convenient means of communication with a physician's office, there are many barriers to its use. Eysenbach has written a thorough review of the potential problems of liability and time pressures [11]. While Katz and his colleagues found no time-savings when email was used as a communication tool, it may well be that it could be an effective tool in some rural settings [12]. Other researchers have also suggested that patient satisfaction and participation in their health care can be increased by use of the Internet by patients [13].

The findings of this survey must be interpreted with caution. While very few patients refused to complete the survey, the patient sample is a convenience sample not a total sample and not a random sample of our patients. With only 46% of our sample (191 patients) having access to the Internet, generalization should be cautious pending replication in a larger sample. The survey instrument did not include questions about readiness to use a touch tone phone for completing a symptom questionnaire by patients, and this is a limitation to the study.

## Conclusion

Our findings suggest that either touch-tone telephone or Internet-based computer methods might be used to collect home-based symptom ratings for cancer patients in both urban and rural centers. While access to technologies is adequate, acceptance and usability of such a system remains to be demonstrated. Patient preference for a telephone-based or Internet-based system can be definitively ascertained only after patients use both systems. With lack of interest being the most common barrier to Internet access, education and "get acquainted" programs for patients who do not have Internet access may be warranted. Alternatively, since many patients prefer touch tone telephone for symptom reporting, the use of IVR (Interactive Voice Recording) technology provides another way for symptom reporting, coupled with centralized nurse care management of cancer-related symptoms. Indeed, we are proceeding to test this in a study in which cancer patients will have an option of home-based symptom monitoring by either IVR or the internet coupled with centralized nurse care management of cancer-related symptoms. Patient resource centers with Internet access in outpatient clinics may be another mechanism to consider.

## Competing interests

The author(s) declare that they have no competing interests.

## Authors' contributions

MA: Assisted with data analysis and interpretation of results. Reviewed, corrected and submitted the final manuscript.

DET: Designed the project including the questionnaire, obtained IRB approval, supervised completion of the survey, assisted with data analysis and writing the final manuscript.

DB: Coordinated data collection from clinic sites and compiled survey results.

KK: Assisted with data analysis, interpretation of results, in addition to review, preparation and submission of the final manuscript.

AP: Carried out the data analysis and assisted with interpretation of results in addition to preparation of the methods and results sections of the manuscript.

SE: Assisted in site recruitment and review of the manuscript.

WMD: Assisted in site recruitment and review of the manuscript.

## Pre-publication history

The pre-publication history for this paper can be accessed here:



## Figures and Tables

**Table 1:** Patient characteristics by urban vs. rural setting

	**Total Sample (n = 416)**	**Urban (n = 186)**	**Rural (n = 230)**	**P-value**
Mean Age	62.7	60.7	64.3	0.013

Gender				0.924
% Female	56.7	57.0	56.5	
% Male	43.3	43	43.5	

Ethnicity				<0.001
% Caucasian	88.6	76.2	98.7	
% Non-Caucasian	11.4	23.8	1.3	

Employment				0.154
% Currently employed	29.1	32.6	26.2	
% Unemployed	70.9	67.4	73.8	

Annual Income				0.155
% < 20 K	31.3	27.3	35.1	
% 20 – 50 K	42.5	42.2	42.7	
% > 50 K	26.2	30.4	22.2	

Education				<0.001
% 8^th ^grade or less	6.3	4.3	7.8	
% Some high school	14.5	11.9	16.5	
% High school or GED	41.5	32.4	48.7	
% Any college	37.8	51.4	27.0	

**Table 2:** Comparison of patient characteristics by E-mail / Internet access

**Patient Characteristic**	**No Email / Internet (n = 219)**	**Email / Internet (n = 191)**	**P-value**
Mean Age	69.1	55.3	0.013

Gender			0.351
% Female	59.0	54.5	
% Male	41	45.5	

Ethnicity			0.150
% Caucasian	91.6	87.2	
%Non-Caucasian	8.4	12.8	

Employment			<0.001
% Currently employed	10.5	50.8	
% Unemployed	89.5	49.2	

Setting			<0.001
% Urban	36.5	53.9	
% Rural	63.5	46.1	

Annual Income			<0.001
% < 20 K	52.4	9.8	
% 20 – 50 K	38.0	47.0	
% > 50 K	9.6	43.3	

Education			<0.001
% 8^th ^grade or less	10.4	1.1	
% Some high school	20.8	6.8	
% High school or GED	49.3	33.0	
% Any college	19.5	59.2	

**Table 3:** Logistic regression results for E-mail / Internet access^1^

**Patient Characteristic**	**Odds Ratio**	**95% CI**
Rural	0.64	(0.35, 1.19)

Age	0.95	(0.93, 0.97)

Female	0.65	(0.37, 1.17)

Caucasian	1.70	(0.65, 4.40)

Currently employed	2.19	(1.06, 4.53)

Annual Income		
< 20 K	1.00	
20 – 50 K	4.54	(2.15, 9.61)
> 50 K	5.98	(2.43, 14.75)

Education		
8^th ^grade or less	0.17	(0.02, 1.52)
Some high school	0.21	(0.07, 0.59)
High school or GED	0.46	(0.25, 0.87)
Any college	1.00	

^1 ^All other variables significant at the univariate level were included in the model and found not to be significant
